# Psychosocial Working Conditions and Social Participation. A 10-Year Follow-Up of Senior Workers

**DOI:** 10.3390/ijerph18179154

**Published:** 2021-08-30

**Authors:** Pia Hovbrandt, Per-Olof Östergren, Catarina Canivet, Maria Albin, Gunilla Carlsson, Kerstin Nilsson, Carita Håkansson

**Affiliations:** 1 Division of Occupational and Environmental Medicine, Department of Laboratory Medicine, Lund University, 22363 Lund, Sweden; Maria.Albin@med.lu.se (M.A.); kerstin.nilsson@med.lu.se (K.N.); carita.hakansson@med.lu.se (C.H.); 2 Occupational Therapy and Occupational Science Research Group, Department of Health Sciences, Lund University, 22100 Lund, Sweden; 3 Division of Social Medicine and Global Health, Department of Clinical Sciences in Malmö, Lund University, 22213 Malmö, Sweden; per-olof.ostergren@med.lu.se (P.-O.Ö.); catarina.canivet@med.lu.se (C.C.); 4 Unit of Occupational Medicine, Institute for Environmental Medicine, Karolinska Institute, 17177 Stockholm, Sweden; 5 Active and Healthy Ageing Research Group, Department of Health Sciences, Lund University, 22100 Lund, Sweden; gunilla.carlsson@med.lu.se

**Keywords:** aging, extended working life, decision latitude, health promotion, life-course perspective, work-life balance, retirement, self-rated health, social activities, sustainable working life, work environment

## Abstract

Social participation is important for health, and it is well known that high strain jobs impact negatively on mental and physical health. However, knowledge about the impact of psychosocial working conditions on social participation from a long-term perspective is lacking. The purpose of this study was to investigate the associations between different job types and social participation from a long-term perspective. A comprehensive public health questionnaire “*The Scania Public Health Survey”,* was used, and psychosocial working conditions were measured with a Swedish translation of the Job Content Questionnaire. Based on data from 1098 working respondents aged 55 at baseline and a 10-year follow-up when the respondents were not working, the analyses revealed that social participation varied by job type. Jobs with high decision latitude, as in active and relaxed jobs, seem to predict high social participation, even after cessation of employment. Besides that, the result suggests that high social participation during working life is a predictor of high social participation from a long-term perspective which promotes healthy aging. Incentives for working longer are strongly related to good working conditions. A supportive work environment with possibilities for employees to participate in decision making, i.e., high control, is vital for a sustainable working life. This may contribute to an extended working life and may also support social participation prior to retirement as well as after retirement and thus to healthy aging.

## 1. Introduction

The demographic change with an increased number of older people is an important factor for public health. Previous findings show for example that experiencing greater enjoyment in life may predict more years in good health [1]. However, the findings are still inconsistent, and it is crucial to find predictors for healthy aging. The concept of “healthy aging” concerns several determinants such as personal and behavioral factors and the social as well as the physical environment [2]. Specifically, the importance of having a life-course perspective is emphasized since aging is a lifelong process [3]. Although social participation patterns remain relatively stable through people’s life course, factors in working life might also impact on opportunities for social participation in very old age [4]. Thus, investigating social participation, which may contribute to health and well-being in old age, from a long-term perspective, is important.

Social participation is a broad concept including leisure activities or meeting with friends and consists of interactions with others in society or the community [5]. Several studies have also found how important social participation is for life satisfaction and healthy aging [6,7,8]. Moreover, social participation contributes to both cognitive and physical health in old age [9,10,11,12,13]. Additionally, formal social participation seems to predict higher levels of quality of life and lower levels of depressive symptoms among older people [14]. What older people value and choose to be engaged in often depends on what they have done in the past, according to the Continuity Theory of Aging [15]. Continuity means an adaptive strategy for change in the aging process, promoted by personal preferences and social behavior based on earlier activities. Supporting the continuity theory, a longitudinal study focusing on age-related changes in leisure, including social participation, showed that participation earlier in life was a strong predictor of participation in leisure activities also in late life [16]. Consequently, it could be important to have a repertoire of activities from the past to choose from in old age, when, for example, work tasks must be replaced with other activities [17,18]. Furthermore, social participation contributes to a sense of being included in a social context [17,19,20], and, among very old people, social participation was shown to be preferable and of special importance for their well-being [21]. However, to the best of our knowledge the influence of working conditions on social participation from a long-term perspective beyond working life has not yet been explored. 

The Job Strain Model (JSM) explains the psychosocial aspects of work and proposes four types of psychosocial job exposure: high strain, relaxed, active, and passive [22]. These four job types are combinations of high and low levels of psychosocial job demands and decision latitude. JSM postulates that high strain (high job demands and low decision latitude) increases the risk of ill health, and empirical support for this has been shown in epidemiological studies of, e.g., coronary heart disease, stroke, diabetes, depression, and neck and shoulder disorders (for recent reviews see [23,24,25]. Job strain has also been related to poor survival in a long-term follow-up after retirement [26].

Among people aged 45–64 years, associations between psychosocial work conditions and social participation using the Job Strain Model have been shown [22,27]. More specifically, Lindström [27] found that passive and high strain jobs were negatively associated with social participation, and that active and relaxed jobs were associated with higher levels of social participation. Work stressors among working adults aged 57–65 was also found to be predictors of limited physical functioning 20 years later [28]. Thus, there may be factors in working life that also have effects on social participation in later life.

Summing up, several studies found that social participation is important for health [6,8,11,13,29], and that high strain jobs impact negatively on mental and physical health [24,25]. However, knowledge about the impact of job strain on social participation from a long-term perspective is lacking. The Scania Public Health Cohort, with 10-year follow-up data, provides a unique opportunity to assess social participation and earlier work-related determinants. In line with previous findings in a cross-sectional study design [27], we hypothesize that there are associations between psychosocial work conditions and social participation from a longitudinal perspective. More precisely, our hypothesis is that low decision latitude, passive job, and job strain are negatively associated with high social participation, and that high decision latitude, active job, and relaxed job are associated with higher levels of social participation.

The purpose of the current study was to investigate the associations between psychosocial working conditions and social participation from a long-term perspective. More specifically the study aimed

- to investigate whether psychosocial demands and their combinations predict social participation among 55-year or older working people in a 10-year follow-up when they were not working.
- to investigate if high decision latitude was associated with social participation at baseline and predicted high social participation at follow-up.

## 2. Material and Methods

### 2.1. Sample and Settings

Comprehensive public health questionnaires, “*The Scania Public Health Survey”,* were sent out, by post, in 2000, 2005, and 2010 to a non-proportional geographically stratified sample of inhabitants in 33 municipalities of the county of Scania in the south of Sweden [30]. These individuals were randomly selected from the population register, such that equal representation was achieved from all 33 municipalities in the region of Scania, Sweden. Details according to design have been described elsewhere [30]. In total, 24,922 subjects born 1919–1981 (age 18–80) were asked to participate in 2000 and of these 13,604 responded (58% response rate). In 2010, an identical questionnaire was sent out to the 12,117 respondents from the first wave who were still alive and living in Scania, which was responded to by 9103 subjects (75% response rate).

**Figure 1:**
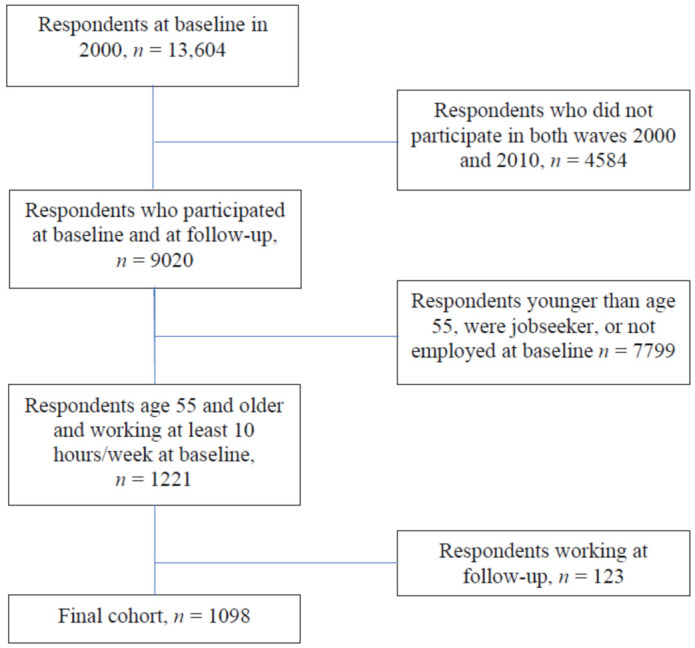
Flow chart of the study sample.

In the present study we included respondents who were 55+ and still working at least 10 h/week at baseline in 2000 and who did not work at follow-up in 2010. The final cohort ended up being 1098 respondents of whom 51% were men and 49% were women (Figure 1).

### 2.2. Outcome Variables

Social participation (during the past year) describes how actively a person has taken part in activities in society. The social participation variable consisted of 13 items: participation in study circle/course at work, study circle/course at leisure time, union meeting, meeting of other organization, theatre/cinema, arts exhibition, church, sports event, had written letter to editor of a newspaper/magazine, demonstration of any kind, visited public event (night club, dance or similar), larger family gathering, or been at a private party. Items were dichotomized (yes/no) and summed up, and if three or less were indicated, the social participation of that person was classified as low, and if four or more were indicated, the social participation of that person was classified as high [31]. This question has been used in Sweden since 1960s and has been validated in an earlier study [32].

### 2.3. Exposure Variable

Psychosocial working conditions were measured with a Swedish translation of the original Job Content Questionnaire (JCQ) [33]. JCQ is based on the JSM [34] and was further developed [22] with a focus on psychosocial demands and control. High demands refer to intensive or rapid work where the employee may experience conflicting demands. Job control refers to the degree of decision-making authority and skill discretion of the employee, i.e., decision latitude. The JCQ items consist of 14 statements where respondents were asked to either agree or disagree on a four level Likert scale (1–4). Thus, the answer is based on the individual’s own experience of demands and control in the working environment. Consequently, there could be variations in the same profession. Two continuous variables reflecting psychosocial job demands and decision latitude were thus created, and both were dichotomized at the median level. Following the demand-control model, four different job types were defined by combining psychosocial demands and decision latitude. That is, high strain job is a combination of high demands and low decision latitude, *relaxed* job is a combination of low demands and high decision latitude, *active* job is a combination of high demands and high decision latitude, and passive job is a combination of low demands and low decision latitude.

### 2.4. Other Baseline Characteristics

Demographic characteristics considered sex, married/cohabitating versus single, and length of education (dichotomized into 12 years and less, corresponding to primary and secondary school, vs. 13 years or more corresponding to university). 

*Financial stress* was captured by the question “How often during the past 12 months have you had difficulties paying your bills (rent, electricity, telephone, mortgage, insurance, etc.)?” with response alternatives “Every month”, “About half of the months”, “A few times” and “Never”. The answer was considered as financial stress if the respondent had answered “Every month” or “About half of the months”, and as “No financial stress” if the answer was “A few times” or “Never”.

To capture the family situation the question was posed, “Do you have any old or sick relative that you need to help, refer to or care for?” with the response alternatives yes or no.

Physical activity was measured by a single question asking about leisure time activity (household work excluded) with the response alternatives: mostly sedentary leisure time activities, moderate leisure time physical activities, regular exercise, hard or competitive sports/training regularly or several times a week. Answers were dichotomized, as physically active (last three alternatives) vs. Not physically active (first alternative). 

Self-rated health was measured with the question, “In general, how do you rate your current health status” with five response alternatives “Excellent”, “Good”, “Fair”, “Bad”, and “Very bad” [35,36,37]. This single question is considered to be the most reliable and valid item estimate of the self-rated health status [38]. Answers were dichotomized as “Good self-rated health” if the respondent had answered “excellent” and “good”, and Poor self-rated health if the answer was “fair”, “bad”, and “very bad” in any of the two waves 1999 and 2010.

### 2.5. Statistical Analysis

Kruskal Wallis test was used to detect differences between the four job types (high strain, relaxed, active, and passive) in social participation rates at baseline and McNemar’s test to detect within each job type group changes in social participation rates between baseline and follow-up 10 years later. Bivariate logistic regression was used to test whether the potential confounders, sex, self-rated health, marital status, not caring for a sick relative, education level, financial stress, and physical activity at baseline, were associated with social participation at follow up. Thereafter, a stepwise multivariate logistic regression analysis was performed to test if high decision latitude at baseline was associated with high social participation at baseline and follow-up. The model was adjusted for the confounders, whose *p*-values in the bivariate logistic regression analyses were <0.10, i.e., good self-rated health, not caring for a sick relative, high educational level, and physically active. Low decision latitude with the lowest social participation rates was selected as the reference category. 

To test for a possible effect modification, i.e., the effect of having two factors worse than additive, a synergy index (SI) was calculated as proposed by Rothman [39]. The following algorithm was used where SI = 1 meant no additive effect, SI > 1 meant a signified synergistic effect, and SI < 1 meant an antagonistic effect.

$$\mathrm{SI}=\frac{\mathrm{RR}\left(\mathrm{AB}\right)\;–\;1}{\left[\mathrm{RR}\;\left(\mathrm{Ab}\right)-1\right]+\left[\mathrm{RR}\left(\mathrm{aB}\right)-1\right]\;}$$

RR = risk ratio; Ab = exposed to one of the factors; aB = exposed to the other factor; AB = exposed to both factors.

The two factors included in this calculation were self-rated health and educational level at baseline. The level of statistical significance was set at *p* < 0.05. The statistical analyses were conducted with SPSS, version 24 (IBM Corp., Armonk, NY, USA).

### 2.6. Ethics

The study was conducted in accordance with the Helsinki Declaration and The Regional Ethical Review Board in Lund approved the study (2016/720).

## 3. Results

### 3.1. Descriptive Results

The mean age of the respondents was 58 years and the oldest individual, who still was working, was 76 years of age at baseline. As shown in Table 1, more than 80% of the respondents in the study were married or cohabiting. Notably, very few of the respondents reported financial stress during the past 12 months. Additionally, very few reported that they were helping a sick or old relative. More than half of the respondents reported good self-rated health, and a quite high proportion reported that they were physically active in their leisure time. Regarding job type, about one third in this study had a passive job (low demands/low decision latitude), almost one fifth of the respondents had a relaxed job (low demands/high decision latitude), an active job (high demands/high decision latitude), or a high strain job (high demands/low decision latitude), respectively.

### 3.2. Variations in Social Participation between Different Job Type 

As can be seen in Table 2, social participation varied by job type. At baseline, high social participation was most common in the active group (91%), followed by 89% in the relaxed group, 82% in the high strain group, and 78% in the passive group; differences between groups were statistically significant (*p* < 0.001). Regarding the within-group evolution in social participation levels between baseline (year 2000) and follow-up (year 2010), social participation decreased in all groups (*p*-levels varying between 0.004 and <0.001). Differences between job type and high social participation at follow up were statistically significant between active and high strain group (*p* < 0.001), active and passive group (*p* < 0.001), relaxed and passive group (*p* < 0.001), and relaxed and high strain group (*p* < 0.001) but not between any other groups. 

### 3.3. Associations between Potential Confounders and High Social Participation

As can be seen in Table 3, the baseline variables associated with high social participation at follow-up were good self-rated health, not caring for sick relative, high educational level, and physically active. Moreover, the strongest predictor of high social participation at follow-up was high social participation at baseline, OR 8.37 (5.84–11.99) (not in tables). 

High decision latitude, physically active, as well as high educational level were associated with high social participation in all models at baseline (Table 4). 

High decision latitude at baseline predicted high social participation at 10-year follow-up. In this model, physically active, good self-rated health at both occasions, high educational level, and not caring for a sick relative were also significant. However, when adjusting for high social participation at baseline high decision latitude, physically active and not caring for a sick relative were no longer significant. High social participation became then the most significant predictor in the model.

We also examined the synergistic effect between latitude at work and self-rated health and educational level on social participation respectively. Not surprisingly, the prevalence of social participation was highest among workers with high decision latitude and good self-rated health and lowest among workers with low decision latitude and poor self-rated health (Table 5). The synergy index between high decision latitude at work and self-rated health on social participation was 2.1, which means that the positive factors reinforce each other’s effect.

Workers with high decision latitude and high educational level showed the highest prevalence of social participation although they did not differ much from workers with low decision latitude and high educational level (Table 6). 

According to educational level the synergy index was 0.66 indicating no additive effect between the positive factors, i.e., high decision latitude and high educational level. This means that the presence of both high decision latitude and high educational level reduced the risk of their separate effects on high social participation. 

## 4. Discussion

### 4.1. Main Findings 

In this study, we investigated the associations between psychosocial working conditions and social participation from a long-term perspective. We firstly tested whether psychosocial demands and their combinations predicted social participation among 55-year or older working people in a 10-year follow-up when they were not working. The result showed that social participation varied by job type, which supports our hypothesis that high decision latitude, active job, and relaxed job should be associated with higher levels of social participation. Thus, it seems like high decision latitude predicts high social participation, even after cessation of employment.

Our findings additionally showed that an unwanted effect of low decision latitude was low social participation. Decision latitude has increasingly been shown to also predict other behavioral patterns of importance for health and well-being, i.e., to be a cause behind the causes [40]. The status syndrome points to the social ingredients of health and that “*the major determinants of health are social”* ([40] p. 1103). Although work gives structure in daily life and contributes to both physical and mental well-being, work may also be stressful. Specifically, circumstances without control in erratic situations, without social support, and without recovery can be very stressful and thus have a negative impact on health [40]. This leads to leisure time physical activity declining among those with low job control [41]. The chance for developing a healthy lifestyle was more likely among those with high decision latitude than for those with low decision latitude [42]. 

Moreover, when we adjusted for social participation at baseline, this was the strongest predictor of social participation at follow-up. This may be understood based on the Continuity Theory [15,43], suggesting that older people are motivated to use their past experiences that worked well to shape their future life course. Thus, older people conceptualize their past as a great resource that influences adaptations to new situations. That is, those with high social participation during working life in the present study could more easily adapt to a situation as retired with continuing social participation. However, in the present study, only the amount of social participation the respondents participated in was included. Regardless, this is in line with previous studies testing the Continuity Theory, which tended to focus on older people’s activity patterns over time [16,44]. These studies also lack information on how respondents value different activities. Among older workers it was found that high workload, i.e., high strain earlier in middle-age, hindered them from valuable activities such as social participation [20]. A balanced life with possibilities for a mix of different activities besides work seems to be important for a sustainable working life that may also contribute to healthy aging. 

Since continuity is not only about what older people do but also and, maybe more importantly, about the meaning behind their participation [45], further studies are needed to explain associations between work-related factors and the meaning in social participation for older people to connect the results more fully to Continuity Theory. 

In line with previous findings, our study confirms how the risk associated with passive and high strain job in midlife also impacts negatively on health factors in old age. For example, longitudinal studies suggested that midlife work stressors were associated with more musculoskeletal pain and mobility problems [28,45,46,47,48] and dementia after retirement age [49]. In a recent study with a focus on passive jobs and high strain jobs in late midlife, it was found that high strain jobs among women and passive jobs among men were associated with decreased physical functioning 20 years later [28]. Thus, it is obvious that psycho-social work conditions such as passive and high strain jobs have negative consequences from a long-term perspective and the present study confirms that this is also true according to social participation. To our knowledge, the current study is the first to show how work-related factors, i.e., especially decision latitude, predicted high social participation in a long-term perspective. Consequently, to strive for a sustainable working life, it is of vital concern to also consider factors in the work environment that may have a positive effect on healthy aging. 

### 4.2. Methodological Considerations

The strength in the present study is the longitudinal design in a national large population with respondents from midlife to old age, and the use of an established psychosocial work condition model, i.e., JSM [33]. Some limitations should also be noted. First, we did not study men and women separately, which should be done in future research. Second, although there is an agreement in research that higher levels of social participation are associated with positive outcomes [50], to count the number of activities is just one aspect of social participation. The number of activities does not say anything of experience of social participation, which is important since it is the meaning of activities that appears to promote health [51]. Future research with qualitative or mixed methods might be applicable to investigate how social participation more deeply might be related to the Continuity Theory of aging. Nevertheless, the findings in the present study gave a picture of how much the respondents participated in the included activities and therefore made it possible to analyze associations with, and predictions of, psychosocial work conditions. Furthermore, another important issue is the possible causality between the variable health and social participation [52]. It may be so that healthier people are more prone to participate in social activities, or the reverse causality that social participation has positive effects on health, or that both social participation and health influence each other [53]. Maybe not very surprisingly, a synergistic effect was found between high decision latitude at work and self-rated health on social participation, while an antagonistic effect between high decision latitude and high educational level was found. 

### 4.3. Possible Implications

This study adds valuable information on the role that midlife psychosocial work environment plays in late life, such as within social participation. For instance, the findings could be used to improve midlife interventions aimed at promoting social participation later in life. Good psychosocial working conditions in a supportive work environment with possibilities for decision making is beneficial not only in working age but also after retirement. Thus, it is of vital concern that both policy makers and employers take actions for a sustainable working life that prevent work injuries and support a balanced working life. 

Considering an aging population in many European countries, an extended working life is of vital concern [54] and, therefore, pension reforms are changing to both restrict early retirement and raise the standard retirement age. However, incentives for working longer are strongly related to good working conditions [55,56] and work strain is also an important risk factor for work ability from a long-term perspective [57,58,59]. Although a previous study showed no associations between physical and psychosocial working conditions and workability [60], another study found that job resources, such as job control, predicted longer time in working life [61]. More and more people actually work beyond the retirement age already today [62], but a large proportion still retires before the statutory retirement age due to work-related risk factors [54,63]. Consequently, an extended working life is only possible with measures that contribute a sustainable working life [64]. Thus, a life-course perspective with measures for a sustainable working life with possibilities for, for example, social participation, is important. A starting point for such a measure could be the theoretical swAge-model [65,66], focusing measurements on health effects associated with working environment, for personal financial security, for social inclusion and social support in the work situation, and for creativity and intrinsic work motivation [67]. A supportive work environment with an organization that encourage employees to participate in decision making, i.e., high control, are vital for a sustainable working life. This may contribute to an extended working life and may also support social participation prior to retirement as well as after retirement, which is an important factor for healthy aging.

## 5. Conclusions

Previous research with long term perspectives has mainly focused on physical functioning and not on other aspects of daily life, such as social dimensions, that are important for healthy aging. In particular, high decision latitude predicted higher levels of social participation while low decision latitude may reduce the chances for active aging. This study adds valuable information on the role that midlife psychosocial work environment plays in late-life, here social participation. For instance, it could potentially be used to improve midlife interventions aimed at promoting social participation later in life. 

## Figures and Tables

**Table 1:** Sociodemographic variables, and psychosocial working conditions at baseline, *n* = 1098.

Characteristics	*n* of Respondents (Missing)	%
Sex		
Men	563	51
Women	535	49
Married/cohabitating	16	2
Yes	894	81
No	188	17
Education	34	3
12 years or less	653	60
13 years or more	411	37
Financial stress	11	1
Yes	19	2
No	1068	97
Self-rated health	(11)	1
Good	669	61
Poor	418	38
Helping old or sick relative	(18)	2
Yes	287	26
No	793	72
Physical activity	(31)	3
Yes	934	85
No	133	12
Job type	(166)	11
Passive	363	33
Relaxed	204	19
Active	227	20
High strain	188	17

**Table 2:** Social participation at baseline and follow-up, in four job type groups, *p* values for change in social participation between 1999 and 2010, *n* = 982.

	Passive, *n* = 363 (%)		*p* Value	Relaxed, *n* = 204 (%)		*p* Value	Active, *n* = 227 (%)		*p* Value	High Strain, *n* = 188		*p* Value
	2000	2010		2000	2010		2000	2010		2000	2010	
High	282 (78)	227 (62)	<0.001	181 (89)	162 (79)	0.004	206 (91)	177 (78)	<0.001	155 (82)	119 (63)	<0.001
Low	81 (22)	136 (38)		23 (11)	42 (21)		21(9)	50 (22)		33 (18)	69 (37)	

**Table 3:** Associations between potential confounders, measured at baseline, and high social participation at the 10-year follow up.

Potential Confounders	OR (95% CI)
Female gender	1.14 (0.88–1.48)
Good self-rated health	**2.11 (1.62–2.74)**
Married/cohabiting	1.25 (0.90–1.75)
Not caring for a sick relative	**1.44 (1.06–1.96)**
High education level	**3.48 (2.56–4.73)**
Financial stress	0.60 (0.24–1.52)
Physically active	**2.40 (1.66–3.48)**

Note: Variables associated with high social participation at follow-up in bold text.

**Table 4:** Associations between high decision latitude at baseline, as defined by the demand-control model, and high social participation at baseline and at the 10-year follow up, respectively (*n* = 982).

	2000					
Variables	Model 1OR (95% CI)	Model 2	Model 3	Model 4	Model 5	Model 6
High decision latitude	2.29 (1.58–3.33)	2.24 (1.53–3.27)	2.12 (1.45–3.10)	1.59 (1.06–2.38)	1.58 (1.05–2.37)	
Physically active		2.44 (1.55–3.86)	2.23 (1.39–3.58)	2.08 (1.27–3.39)	2.08 (1.27–3.41)	
Good self-rated health 1999			1.32 (0.90–1.94)	1.20 (0.80–1.80)	1.22 (0.81–1.84)	
High educational level				3.08 (1.93–4.92)	3.01 (1.88–4.81)	
Not caring for a sick relative					1.11 (0.72-1.70)	
	2010					
High decision latitude	2.18 (1.64–2.91)	2.13 (1.59–2.86)	2.01 (1.49–2.70)	1.53 (1.12–2.10)	1.49 (1.09–2.05)	1.37 (0.98–1.91)
Physically active		2.38 (1.58–3.58)	1.96 (1.29–2.99)	1.79 (1.16–2.78)	1.78 (1.15–2.77)	1.51 (0.94–2.42)
Good self-rated health 1999–2010			1.90 (1.41–2.55)	1.90 (1.40–2.58)	1.94 (1.42–2.64)	1.92 (1.38–2.66)
High educational level				3.01 (2.14–4.26)	2.98 (2.11–4.21)	2.50 (1.74–3.58)
Not caring for a sick relative					1.43 (1.01–2.03)	1.44 (0.99–2.08)
High social participation at baseline						6.29 (4.15–9.54)

**Table 5:** Interaction analysis with synergy index, regarding latitude at work and self-rated health, both measured at baseline in 1999 and social participation in 2010. Scania Public Health Cohort, *n* = 975.

Decision Latitude and Self-Rated Health in 2000	*n*	% Cases with High Social Participation	OR ^a^	95% CI ^b^	SI ^c^
Low latitude and poor health	176	53	1		
Low latitude and good health	369	68	1.4	0.9–2.1	
High latitude and poor health	90	64	1.7	0.9–3.3	
High latitude and good health	340	82	3.2	1.9–5.4	2.1
	975				

^a^ OR, odds ratio; ^b^ CI, confidence interval; ^c^ SI, synergy index.

**Table 6:** Interaction analyses with synergy index, regarding latitude at work and level of education, both measured at baseline in 1999 and social participation in 2010. Scania Public Health Cohort, *n* = 961.

Decision Latitude and Educational Level in 2000	*n*	% Cases with High Social Participation	OR ^a^	95% CI ^b^	SI ^c^
Low latitude and low educational level	386	56	1		
Low latitude and high educational level	149	83	5.2	2.6–10.6	
High latitude and low educational level	195	71	2.1	1.3–3.3	
High latitude and high educational level	231	85	4.5	2.6–7.9	0.66
	961				

^a^ OR, odds ratio; ^b^ CI, confidence interval; ^c^ SI, synergy index.

## Data Availability

The dataset used and analysed during the current study is available from the corresponding author on reasonable request.
